# Assessment of the Feasibility of automated, real-time clinical decision support in the emergency department using electronic health record data

**DOI:** 10.1186/s12873-018-0170-9

**Published:** 2018-07-03

**Authors:** Warren M. Perry, Rubayet Hossain, Richard A. Taylor

**Affiliations:** 1 Emergency Medicine Department, Yale School of Medicine, 464 Congress Avenue, Suite #260, New Haven, CT 06450 USA; 2 Emergency Department, Yale New Haven Hospital, 20 York Street, New Haven, CT 06510 USA; 3 Yale School of Medicine, Yale New Haven Hospital, 464 Congress Avenue, Suite #260, New Haven, CT 06450 USA

**Keywords:** Clinical decision support, Machine learning, Data quality, Electronic health records

## Abstract

**Background:**

The use of big data and machine learning within clinical decision support systems (CDSSs) has the potential to transform medicine through better prognosis, diagnosis and automation of tasks. Real-time application of machine learning algorithms, however, is dependent on data being present and entered prior to, or at the point of, CDSS deployment. Our aim was to determine the feasibility of automating CDSSs within electronic health records (EHRs) by investigating the timing, data categorization, and completeness of documentation of their individual components of two common Clinical Decision Rules (CDRs) in the Emergency Department.

**Methods:**

The CURB-65 severity score and HEART score were randomly selected from a list of the top emergency medicine CDRs. Emergency department (ED) visits with ICD-9 codes applicable to our CDRs were eligible. The charts were reviewed to determine the categorization components of the CDRs as structured and/or unstructured, median times of documentation, portion of charts with all data components documented as structured data, portion of charts with all structured CDR components documented before ED departure. A kappa score was calculated for interrater reliability.

**Results:**

The components of the CDRs were mainly documented as structured data for the CURB-65 severity score and HEART score. In the CURB-65 group, 26.8% of charts had all components documented as structured data, and 67.8% in the HEART score. Documentation of some CDR components often occurred late for both CDRs. Only 21 and 11% of patients had all CDR components documented as structured data prior to ED departure for the CURB-65 and HEART score groups, respectively. The interrater reliability for the CURB-65 score review was 0.75 and 0.65 for the HEART score.

**Conclusion:**

Our study found that EHRs may be unable to automatically calculate popular CDRs—such as the CURB-65 severity score and HEART score—due to missing components and late data entry.

**Electronic supplementary material:**

The online version of this article (10.1186/s12873-018-0170-9) contains supplementary material, which is available to authorized users.

## Background

The use of big data and machine learning within clinical decision support systems (CDSSs) has the potential to transform medicine through better prognosis, diagnosis and automation of tasks [1, 2]. Real-time application of machine learning algorithms, however, is dependent on data being present and entered prior to, or at the point of, CDSS deployment [3]. While simple CDSSs may request the user to enter in data necessary to run the algorithm, this process becomes infeasible when tens or even hundreds of data elements are needed, thus requiring some form of automated capture and real-time integration of data from the electronic health record (EHR) [4].

The emergency department (ED), with a highly condensed time frame for decision making, represents a unique and challenging environment for CDSS deployment [5]. In the ED, patient medical problems are often acute with limited supporting information already present in the EHR, most of the information necessary for decision making is generated within a few hours, and recording of parts of this information in the form of provider notes may be delayed until well after the patient has left the department [6]. Currently, there is limited knowledge on when components necessary for calculation of CDSS in the ED are available in the EHR, what data type (structured vs unstructured) they exist in, and data completeness for specific algorithms [7].

Our study, therefore, aimed to examine the EHR data entry process for two common, simple clinical decision rules (CDRs) as a first step in assessing the potential feasibility of constructing more complex automated, real-time clinical decision support systems in the ED. Specifically, we examined whether the CDRs were calculable using EHR data, how the individual CDR components are recorded (structured versus unstructured data), and the timeliness of data entry.

## Methods

### Study design

This was a retrospective study of ED visits between 1/1/2015 and 1/1/2016. The study was approved by the institutional review board.

### Study site

The study involved five different emergency department sites with combined annual visits > 250,000. All of the study sites are part of a larger hospital healthcare system with a single EHR vendor. The hospital system serves both an urban and suburban patient population. The sites are staffed with certified nursing assistants, midlevel providers, residents, and attending physicians.

### Identification of clinical decision rules

The research team contacted the creators of the website, www.MDcalc.com, to acquire a list of the calculators used in the Emergency Medicine section. This table included the full list of calculators and the percentage each calculator composed of the total searches for all calculators in the 3 months prior to July 16, 2015. From this list of calculators, we ordered them from highest to lowest percentage of searches. We defined a clinical decision rule as a “clinical tool that quantifies the individual contributions that various components of the history, physical examination, and basic laboratory results make toward the diagnosis, prognosis, or likely response to treatment in a patient” [8]. A calculator from the list was eligible for selection if it met this definition and excluded if it was not created for the purpose of being a CDR, was targeted to a population less than 18 years of age, had no diagnostic significance, no prognostic significance for a condition or treatment, or cannot be calculated in the emergency department. From this definition, the research team identified the top 10 clinical decision rules, and randomly selected two from the list to investigate, the CURB-65 score and HEART score.

### Patient chart selection and review

The calculated sample size of each CDR, assuming 70% structured data, 95% confidence intervals for a 7.5% margin of error was 140 charts. We selected 145 charts to review for both the CURB-65 score and HEART score. In order to identify charts where the CDRs would be used, the team performed a keyword search to make a list of ICD-9 codes that corresponded with the diagnoses targeted by each CDR (Additional file 1: Table S1). From this list, the team performed a database query for all emergency department diagnoses within the study period that matched the ICD-9 codes identified for each CDR. Prior to full review, the team randomly selected and reviewed 10 separate charts from each CDR list to ensure the chosen ICD-9 codes appropriately selected patients with medical presentations applicable to their corresponding CDR.

### Data collection

Patient charts were stored in a database (FileMaker, Inc., FileMaker International) as multiple iterations with noted timestamps to document changes made to the patient chart (i.e. every time a chart was modified a new timestamped instance was generated). The team excluded a chart if the chart had more iterations than is supported by the query text field and was therefore truncated. In the analysis, the charts were reviewed to categorize the individual CDR components as structured or unstructured data. The team defined structured data as any data with designated fields containing discrete data elements within the electronic health record. All other data was considered unstructured. Each element documented, had its corresponding timestamp of when it appeared in the EHR documented as well. The team documented the portion of charts with all structured data components required to calculate each corresponding CDR. Furthermore, the total time from triage to disposition, and the portion of charts with all data components entered as structured data prior to the patient’s departure from the emergency department were obtained. All chart reviewers had at least 5 months of experience using EPIC and went through training to ensure all members understood what data is structured versus unstructured. The team agreed on a guide of instructions for both the CURB-65 score and HEART score CDRs to standardize the review (Additional file 2: Appendix B).

### Data analysis

Results were analyzing using standard descriptive statistics. Box plots were generated for time to date entry of the various components and operational metrics. A kappa score was calculated to assess the interrater reliability among reviewers. All analysis was performed in R (**R** Core Team (2017). **R**: A language and environment for statistical computing).

## Results

A total of 83 calculators were received from the creators of www.MDcalc.com. Seventeen calculators were excluded to reach our final list of ten emergency medicine clinical decision rules. To view a full list of these calculators and why each one was excluded, please refer to Additional file 3: Appendix C. One hundred and forty-five charts were reviewed for the CURB-65 rule and HEART score, each. In the HEART score group, 2 charts had either missing or errant data from the review process and were excluded in the data analysis. The demographic information for the patients is demonstrated in Table 1. The Kappa statistic was 0.75 and 0.65 for the CURB-65 and HEART score respectively. Overall, the HEART score was able to be calculated 67.8% of the time and the CURB-65 rule 26.8% of the time from the provided structured data.

**Table 1:** Study demographics

	CURB-65^a^ Score	HEART^b^ Score
N	145	143
Median age	64.00	51.00
Male n (%)	68 (46.9)	54 (37.8)
Ethnicity n (%)		
Hispanic or Latino	26 (17.9)	28 (19.6)
Non-Hispanic	118 (81.4)	115 (80.4)
Unknown	1 (0.7)	0 (0.0)
Race n (%)		
Asian	3 (2.1)	0 (0.0)
Black or African American	21 (14.5)	34 (23.8)
Hispanic/Latino	1 (0.7)	2 (1.4)
Not documented	1 (0.7)	2 (1.4)
Other	26 (17.9)	23 (16.1)
Patient refused	2 (1.4)	1 (0.7)
White or Caucasian	91 (62.8)	81 (56.6)

^a^Confusion, Urea, Respiratory rate, Blood pressure, Age ≥ 65

^b^History, Electrocardiogram, Age, Risk factors, Troponin

**Fig. 1:**
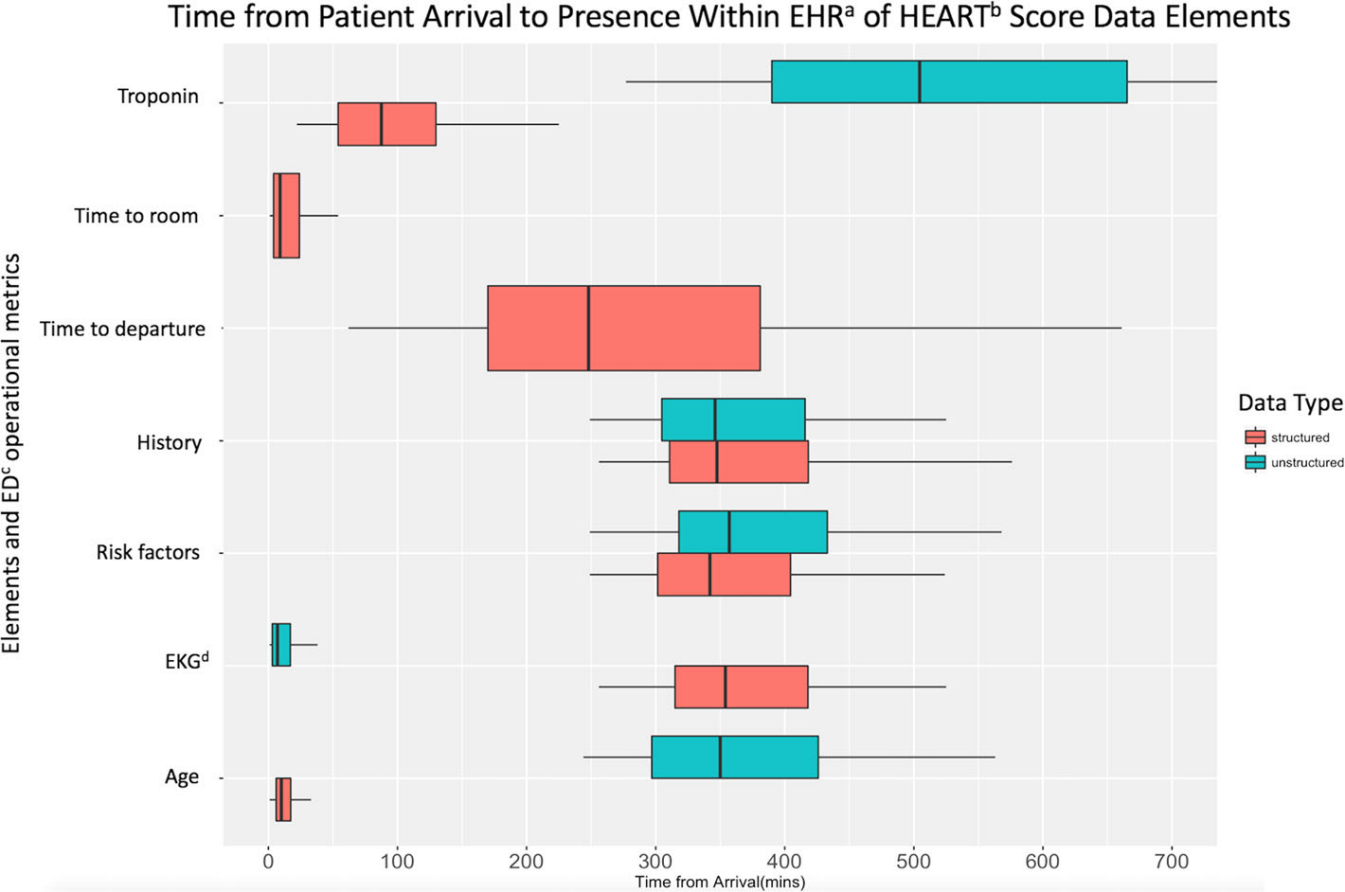
Time from Arrival to Presence Within EHR^a^ of HEART^b^ Score Data. The above figure is a box and whiskers plot representation of Table 3 data from the HEART score. ^a^ electronic health record. ^b^ History, Electrocardiogram, Age, Risk Factors, Troponin. ^c^ emergency department. ^d^ electrocardiogram

**Fig. 2:**
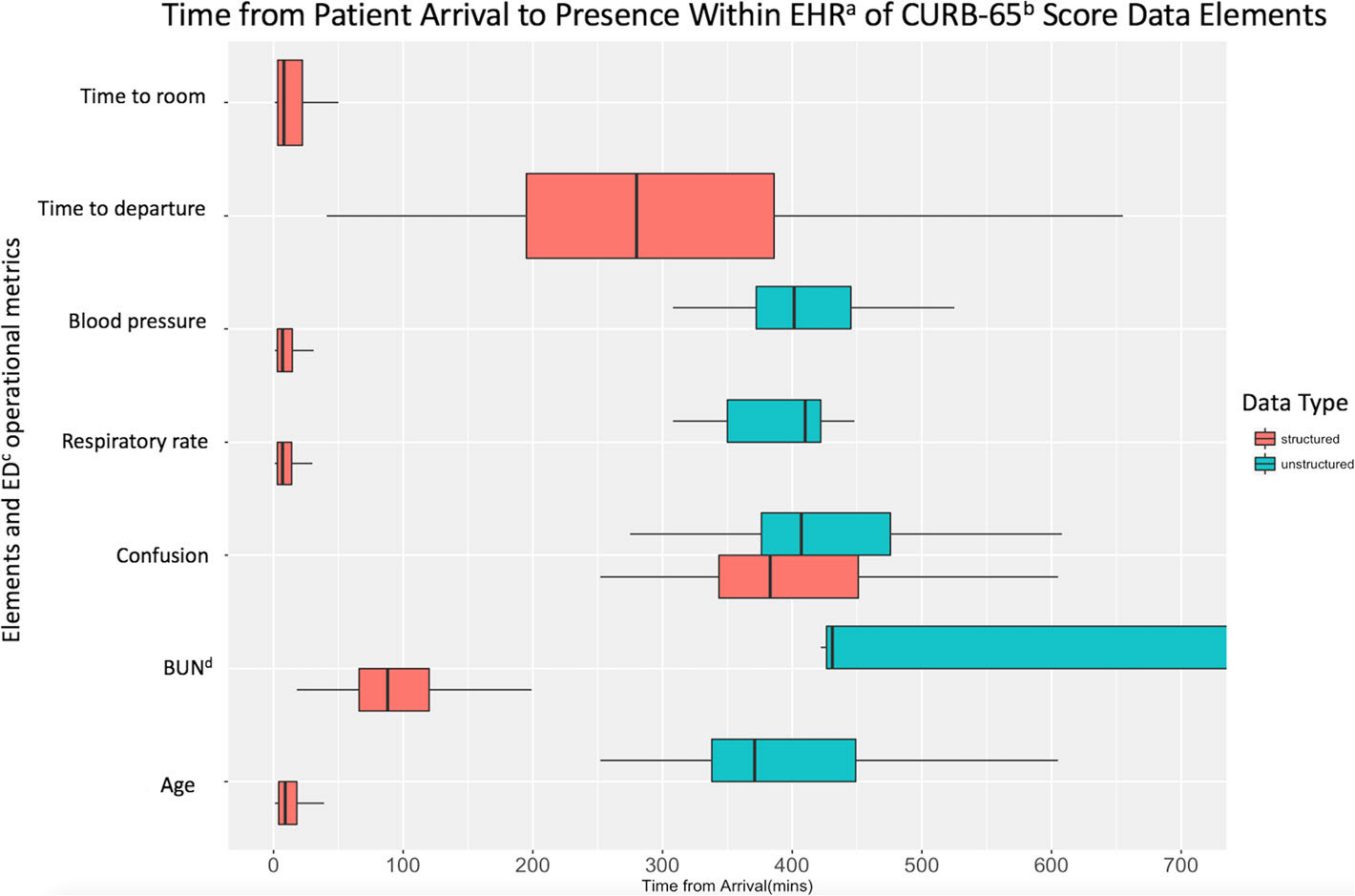
Time from Arrival to Presence Within EHR^a^ of CURB-65^b^ Score Data. The above figure is a box and whiskers plot representation of Table 3 data from the CURB-65 score. ^a^ electronic health record. ^b^ Confusion, Urea, Respiratory Rate, Blood Pressure, Age ≥ 65. ^c^ emergency department. ^d^ blood urea nitrogen leve

Percentage documentation of structured and unstructured date elements for the HEART and CURB-65 score are presented in Table 2. Median times and interquartile ranges for each unstructured and structured data components of each rule are presented in Table 3 and Figs. 1 and 2. The median time from triage to a disposition was 280 (IQR 195–386] and 248 (IQR 170–381] minutes for the CURB-65 score and HEART score, respectively. Approximately 21% of the CURB-65 score patients and 11% of the HEART score patients had all their CDR components entered prior to their departure from the emergency department.

**Table 2:** Portion of data elements documented as structured vs. Unstructured

CURB-65^a^ Score			HEART^b^ Score		
Elements	Structured ^c^	Unstructured ^c^	Elements	Structured ^c^	Unstructured ^c^
Confusion	141 (97.2)	22 (15.2)	Age	143 (100.0)	75 (52.4)
Blood urea nitrogen	117 (80.7)	3 (2.1)	Risk factors	127 (88.8)	87 (60.8)
Respiratory rate	145 (100.0)	9 (6.2)	History	128 (89.5)	128 (89.5)
Blood pressure	144 (99.3)	10 (6.9)	Electrocardiogram	66 (46.2)	141 (98.6)
Age	145 (100.0)	88 (60.7)	Troponin	120 (83.9)	54 (37.8)
All structured elements documented (%)	26.8		67.8		
All structured elements documented prior to ED^d^ departure (%)	21		11		
Kappa statistic	0.75		0.65		

^a^Confusion, Urea, Respiratory rate, Blood pressure, Age ≥ 65

^b^History, Electrocardiogram, Age, Risk factors, Troponin

^c^Results reported as n (%)

^d^Emergency department

**Table 3:** Median times and interquartile ranges for elements documentation^a^

CURB-65^b^ Score			HEART^c^ Score		
Elements	Structured	Unstructured	Elements	Structured	Unstructured
Confusion	383.00 [343.50, 451.00]	407.00 [376.25, 475.75]	Age	10.00 [6.00, 17.25]	350.00 [297.00, 426.00]
Blood area nitrogen	88.00 [66.00, 120.00]	431.00 [426.50, 2401.00]	Electrocardiogram	354.00 [315.00, 418.00]	7.00 [3.00, 17.00]
Respiratory rate	7.00 [3.00, 14.00]	410.00 [350.00, 422.00]	Risk factors	342.00 [301.50, 404.50]	357.00 [318.00, 433.00]
Blood pressure	7.00 [3.00, 14.50]	401.50 [372.25, 445.25]	History	347.50 [310.75, 418.25]	346.00 [304.75, 415.75]
Age	9.00 [4.00, 18.00]	371.00 [338.00, 449.00]	Troponin	87.50 [54.00, 129.75]	504.50 [390.00, 665.25]
Time to departure	280.00 [195.00, 386.00]		248.00 [170.00, 381.00]		

^a^All items are reported in minutes, median time [interquartile range time]

^b^Confusion, Urea, Respiratory rate, Blood pressure, Age ≥ 65

^c^History, Electrocardiogram, Age, Risk factors, Troponin

## Discussion

Automated real-time CDSSs integrating machine learning algorithms trained on big data have the potential to transform healthcare, however necessary data for the algorithms must be present at the time of use, and little is known about how data availability impacts the feasibility of these systems in general, and more specifically within the emergency department. This study examines the data entry process for two popular clinical decision rules, CURB-65 score and HEART score as a first step in assessing the feasibility of more complex CDSSs. Our findings demonstrate that, even for these simple CDRs, the HEART score and CURB-65 rule were only able to be calculated prior to ED departure in 21, and 11% of visits, respectively. For structured data only, regardless of timing, the HEART score was able to be calculated in 67.8% of visits and the CURB-65 rule in 26.8% of visits. Data components that depended on historical information (confusion, risk factors, etc.) or provider entry of data were entered frequently after ED departure.

Prior studies have noted the need for EHR integration of CDSSs, however previous studies examining the ability of CDRs to be automated are limited [7, 9–11]. Aakre et al. [10] investigated the programmability of clinical decision rules in an EHR based on the ability of their components to be extracted from structured data sources or retrieved using advanced technology such as natural language processing (NLP) and Boolean logic text search. Out of the 168 CDRs investigated in this study, only 26 (15.5%) were programmable exclusively using structured objective data elements. If additional advanced technology, such as NLP and Boolean logic text search, were utilized to extract both the structured and unstructured subjective components, this theoretical number increased to 43 (25.6%). This study did not examine the data completeness or timeliness of these CDRs within an existing EHR. Sheehan et al. [7], examined through qualitative analysis the Pediatric Emergency Care Applied Research Network (PECARN) clinical prediction rules for children with minor blunt head trauma and found the need for seamless integration, flowsheets to facilitate data entry, among other workflow, organizational, and human factors impeding automization. Our study adds to this knowledge about the difficulty and planning necessary for CDSSs.

Our findings have important implications for CDSS implementation within the ED. If CDDSs are to be effectively utilized, the data must be available at the point in time the CDSS provides value in the decision-making process. The fact that in our study the CDRs were often unable to be calculated from structured data indicates that for CDSSs to be effective structured fields must be built within the EHR that are designed to capture the components of the CDSSs and/or natural language processing should be utilized. In addition, CDSSs, must take into consideration the timing of data entry. For the two CDRs in this study, most of the delay in calculability resulted from data components collected through the history and physical or were dependent on provider entry of data (e.g. EKG interpretation). This is not surprising as ED providers often get behind in documentation during a shift or wait until after their shifts to complete documentation [6]. This lack of timeliness implies that the performance of CDSSs within the ED will be hampered by data entry and future efforts at CDSSs should consider more effective methods of timely data entry such as speech recognition with natural language processing pipelines.

### Limitations

Our study has several limitations. We only chose two clinical decision rules to examine. While it is possible that other CDRs would have better data-entry, we believe that the themes derived from our results (late entry of historical/physical findings, and overall poor ability to calculate in a timely fashion) would be the same. It is possible that emergency physicians at our site could have used other CDRs relevant to our patient population, but the research team did not observe other CDRs documented as unstructured data in the patient chart. Some of the clinicians may not believe in the value of our selected CDRs; however, we chose our CDRs randomly from a reliable source of the top searched emergency medicine calculators. The guidelines for presence of the data components we used in our study were not strict, but this method optimized the number of components that satisfied our criteria and provided a best-case scenario. Even with these relaxed parameters, the findings show that all components are not documented for the CURB-65 and HEART scores. Lastly, our study had a small sample size of patients and thus we were unable to determine the effects of different ED environments for documentation (e.g., effect of provider type, presence or absence of dictation software).

## Conclusion

Our study assessing the potential feasibility of constructing more complex automated, real-time CDSSs in the ED through examination of the EHR data entry process for two common, simple CDRs demonstrates that the CDRs cannot be calculated reliably during the emergency department visit because of problems with data completeness and timeliness of entry.

## Additional files

Additional file 1: **Appendix A.** Applicable ICD-9 Codes for Clinical Decision Rules. ICD Codes used for identifying cases for chart review. (DOCX 23 kb)

Additional file 2: **Appendix B.** Guideline to the Standard Evaluation of Charts. Outline and set of instructions of how each data element was encoded by the reviewers. (DOCX 15 kb)

Additional file 3: **Appendix C.** Inclusion and Exclusion of Emergency Medicine Calculators. List of Calculators, Clinical Decision Rules from MDCalc. (DOCX 16 kb)

## Abbreviations

CDR: Clinical Decision Rule
CDSSs: Clinical decision support systems
ED: Emergency department
EHR: electronic health records
