# Supplementary material for: Assessment of the Feasibility of automated, real-time clinical decision support in the emergency department using electronic health record data
Source: BMC Emerg Med. 2018 Jul 3;18:19. doi: 10.1186/s12873-018-0170-9 (PMC6029277; doi:10.1186/s12873-018-0170-9)
Supplement: Supplementary file 1 — Appendix A. Applicable ICD-9 Codes for Clinical Decision Rules. ICD Codes used for identifying cases for chart review. (DOCX 23 kb) [file 12873_2018_170_MOESM1_ESM.docx]

| **Appendix A: Table A1- Applicable ICD-9 Codes for Clinical Decision Rules** |  |  |
| --- | --- | --- |
| Clinical Decision Rule | Diagnosis | ICD-9 Code |
| **CURB-65 severity score^a^** | PNEUMONIA DUE TO ADENOVIRUS | 480.00 |
|  | PNEUMONIA DUE TO RESPIRATORY SYNCYTIAL VIRUS | 480.10 |
|  | PNEUMONIA DUE TO PARAINFLUENZA VIRUS | 480.20 |
|  | PNEUMONIA DUE TO SARS-ASSOCIATED CORONAVIRUS | 480.30 |
|  | PNEUMONIA DUE TO OTHER VIRUS NOT ELSEWHERE CLASSIFIED | 480.80 |
|  | VIRAL PNEUMONIA UNSPECIFIED | 480.90 |
|  | PNEUMOCOCCAL PNEUMONIA [STREPTOCOCCUS PNEUMONIAE PNEUMONIA] | 481.00 |
|  | PNEUMONIA DUE TO KLEBSIELLA PNEUMONIAE | 482.00 |
|  | PNEUMONIA DUE TO PSEUDOMONAS | 482.10 |
|  | PNEUMONIA DUE TO HEMOPHILUS INFLUENZAE (H. INFLUENZAE) | 482.20 |
|  | PNEUMONIA DUE TO STREPTOCOCCUS UNSPECIFIED | 482.30 |
|  | PNEUMONIA DUE TO STREPTOCOCCUS GROUP A | 482.31 |
|  | PNEUMONIA DUE TO STREPTOCOCCUS GROUP B | 482.32 |
|  | PNEUMONIA DUE TO OTHER STREPTOCOCCUS | 482.39 |
|  | PNEUMONIA DUE TO STAPHYLOCOCCUS UNSPECIFIED | 482.40 |
|  | METHICILLIN SUSCEPTIBLE PNEUMONIA DUE TO STAPHYLOCOCCUS AUREUS | 482.41 |
|  | METHICILLIN RESISTANT PNEUMONIA DUE TO STAPHYLOCOCCUS AUREUS | 482.42 |
|  | OTHER STAPHYLOCOCCUS PNEUMONIA | 482.49 |
|  | PNEUMONIA DUE TO ANAEROBES | 482.81 |
|  | PNEUMONIA DUE TO ESCHERICHIA COLI [E.COLI] | 482.82 |
|  | PNEUMONIA DUE TO OTHER GRAM-NEGATIVE BACTERIA | 482.83 |
|  | PNEUMONIA DUE TO LEGIONNAIRES' DISEASE | 482.84 |
|  | PNEUMONIA DUE TO OTHER SPECIFIED BACTERIA | 482.89 |
|  | BACTERIAL PNEUMONIA UNSPECIFIED | 482.90 |
|  | PNEUMONIA DUE TO MYCOPLASMA PNEUMONIAE | 483.00 |
|  | PNEUMONIA DUE TO CHLAMYDIA | 483.10 |
|  | PNEUMONIA DUE TO OTHER SPECIFIED ORGANISM | 483.80 |
|  | PNEUMONIA IN CYTOMEGALIC INCLUSION DISEASE | 484.10 |
|  | PNEUMONIA IN WHOOPING COUGH | 484.30 |
|  | PNEUMONIA IN ANTHRAX | 484.50 |
|  | PNEUMONIA IN ASPERGILLOSIS | 484.60 |
|  | PNEUMONIA IN OTHER SYSTEMIC MYCOSES | 484.70 |
|  | PNEUMONIA IN OTHER INFECTIOUS DISEASES CLASSIFIED ELSEWHERE | 484.80 |
|  | BRONCHOPNEUMONIA ORGANISM UNSPECIFIED | 485.00 |
|  | PNEUMONIA ORGANISM UNSPECIFIED | 486.00 |
|  | INFLUENZA WITH PNEUMONIA | 487.00 |
|  | INFLUENZA DUE TO IDENTIFIED AVIAN INFLUENZA VIRUS WITH PNEUMONIA | 488.01 |
|  | INFLUENZA DUE TO IDENTIFIED 2009 H1N1 INFLUENZA VIRUS WITH PNEUMONIA | 488.11 |
|  | INFLUENZA DUE TO IDENTIFIED NOVEL INFLUENZA A VIRUS WITH PNEUMONIA | 488.81 |
| **HEART score^b^** | ANGINA DECUBITUS | 413.00 |
|  | PRINZMETAL ANGINA | 413.10 |
|  | OTHER AND UNSPECIFIED ANGINA PECTORIS | 413.90 |
|  | UNSPECIFIED CHEST PAIN | 786.50 |
|  | OTHER CHEST PAIN | 786.59 |
|  | ACUTE MYOCARDIAL INFARCTION OF ANTEROLATERAL WALL EPISODE OF CARE UNSPECIFIED | 410.00 |
|  | ACUTE MYOCARDIAL INFARCTION OF ANTEROLATERAL WALL INITIAL EPISODE OF CARE | 410.01 |
|  | ACUTE MYOCARDIAL INFARCTION OF ANTEROLATERAL WALL SUBSEQUENT EPISODE OF CARE | 410.02 |
|  | ACUTE MYOCARDIAL INFARCTION OF OTHER ANTERIOR WALL EPISODE OF CARE UNSPECIFIED | 410.10 |
|  | ACUTE MYOCARDIAL INFARCTION OF OTHER ANTERIOR WALL INITIAL EPISODE OF CARE | 410.11 |
|  | ACUTE MYOCARDIAL INFARCTION OF OTHER ANTERIOR WALL SUBSEQUENT EPISODE OF CARE | 410.12 |
|  | ACUTE MYOCARDIAL INFARCTION OF INFEROLATERAL WALL EPISODE OF CARE UNSPECIFIED | 410.20 |
|  | ACUTE MYOCARDIAL INFARCTION OF INFEROLATERAL WALL INITIAL EPISODE OF CARE | 410.21 |
|  | ACUTE MYOCARDIAL INFARCTION OF INFEROLATERAL WALL SUBSEQUENT EPISODE OF CARE | 410.22 |
|  | ACUTE MYOCARDIAL INFARCTION OF INFEROPOSTERIOR WALL EPISODE OF CARE UNSPECIFIED | 410.30 |
|  | ACUTE MYOCARDIAL INFARCTION OF INFEROPOSTERIOR WALL INITIAL EPISODE OF CARE | 410.31 |
|  | ACUTE MYOCARDIAL INFARCTION OF INFEROPOSTERIOR WALL SUBSEQUENT EPISODE OF CARE | 410.32 |
|  | ACUTE MYOCARDIAL INFARCTION OF OTHER INFERIOR WALL EPISODE OF CARE UNSPECIFIED | 410.40 |
|  | ACUTE MYOCARDIAL INFARCTION OF OTHER INFERIOR WALL INITIAL EPISODE OF CARE | 410.41 |
|  | ACUTE MYOCARDIAL INFARCTION OF OTHER INFERIOR WALL SUBSEQUENT EPISODE OF CARE | 410.42 |
|  | ACUTE MYOCARDIAL INFARCTION OF OTHER LATERAL WALL EPISODE OF CARE UNSPECIFIED | 410.50 |
|  | ACUTE MYOCARDIAL INFARCTION OF OTHER LATERAL WALL INITIAL EPISODE OF CARE | 410.51 |
|  | ACUTE MYOCARDIAL INFARCTION OF OTHER LATERAL WALL SUBSEQUENT EPISODE OF CARE | 410.52 |
|  | TRUE POSTERIOR WALL INFARCTION EPISODE OF CARE UNSPECIFIED | [410.60](https://www.cms.gov/medicare-coverage-database/staticpages/icd-9-code-lookup.aspx?KeyWord=true%20posterior%20wall&bc=AAAAAAAAAAAEAA%3d%3d&) |
|  | TRUE POSTERIOR WALL INFARCTION INITIAL EPISODE OF CARE | [410.61](https://www.cms.gov/medicare-coverage-database/staticpages/icd-9-code-lookup.aspx?KeyWord=true%20posterior%20wall&bc=AAAAAAAAAAAEAA%3d%3d&) |
|  | TRUE POSTERIOR WALL INFARCTION SUBSEQUENT EPISODE OF CARE | [410.62](https://www.cms.gov/medicare-coverage-database/staticpages/icd-9-code-lookup.aspx?KeyWord=true%20posterior%20wall&bc=AAAAAAAAAAAEAA%3d%3d&) |
|  | SUBENDOCARDIAL INFARCTION EPISODE OF CARE UNSPECIFIED | [410.70](https://www.cms.gov/medicare-coverage-database/staticpages/icd-9-code-lookup.aspx?KeyWord=subendocardial%20inf&bc=AAAAAAAAAAAEAA%3d%3d&) |
|  | SUBENDOCARDIAL INFARCTION INITIAL EPISODE OF CARE | [410.71](https://www.cms.gov/medicare-coverage-database/staticpages/icd-9-code-lookup.aspx?KeyWord=subendocardial%20inf&bc=AAAAAAAAAAAEAA%3d%3d&) |
|  | SUBENDOCARDIAL INFARCTION SUBSEQUENT EPISODE OF CARE | [410.72](https://www.cms.gov/medicare-coverage-database/staticpages/icd-9-code-lookup.aspx?KeyWord=subendocardial%20inf&bc=AAAAAAAAAAAEAA%3d%3d&) |
|  | ACUTE MYOCARDIAL INFARCTION OF OTHER SPECIFIED SITES EPISODE OF CARE UNSPECIFIED | 410.80 |
|  | ACUTE MYOCARDIAL INFARCTION OF OTHER SPECIFIED SITES INITIAL EPISODE OF CARE | 410.81 |
|  | ACUTE MYOCARDIAL INFARCTION OF OTHER SPECIFIED SITES SUBSEQUENT EPISODE OF CARE | 410.82 |
|  | ACUTE MYOCARDIAL INFARCTION OF UNSPECIFIED SITE EPISODE OF CARE UNSPECIFIED | 410.90 |
|  | ACUTE MYOCARDIAL INFARCTION OF UNSPECIFIED SITE INITIAL EPISODE OF CARE | [410.91](https://www.cms.gov/medicare-coverage-database/staticpages/icd-9-code-lookup.aspx?KeyWord=myocardial%20infarction&bc=AAAAAAAAAAAEAA%3d%3d&) |
|  | ACUTE MYOCARDIAL INFARCTION OF UNSPECIFIED SITE SUBSEQUENT EPISODE OF CARE | [410.92](https://www.cms.gov/medicare-coverage-database/staticpages/icd-9-code-lookup.aspx?KeyWord=myocardial%20infarction&bc=AAAAAAAAAAAEAA%3d%3d&) |
|  | ACUTE CORONARY OCCLUSION WITHOUT MYOCARDIAL INFARCTION | [411.81](https://www.cms.gov/medicare-coverage-database/staticpages/icd-9-code-lookup.aspx?KeyWord=myocardial%20infarction&bc=AAAAAAAAAAAEAA%3d%3d&) |
|  | CARDIAC ARREST | 427.50 |
| ^a^Confusion, Urea, Respiratory rate, Blood pressure, Age ≥ 65 | |  |
| ^b^History, Electrocardiogram, Age, Risk factors, Troponin | |  |
