# Supplementary material for: Assessment of the Feasibility of automated, real-time clinical decision support in the emergency department using electronic health record data
Source: BMC Emerg Med. 2018 Jul 3;18:19. doi: 10.1186/s12873-018-0170-9 (PMC6029277; doi:10.1186/s12873-018-0170-9)
Supplement: Supplementary file 2 — Appendix B. Guideline to the Standard Evaluation of Charts. Outline and set of instructions of how each data element was encoded by the reviewers. (DOCX 15 kb) [file 12873_2018_170_MOESM2_ESM.docx]

**Guidelines to the Standard Evaluation of Charts**

**Heart Score:**

- History-
  - Structured
    - Chest pain documented in History of Present Illness section or review of system section of the electronic health record or “all other systems negative” tagged in the review of systems section of the electronic health record.
  - Unstructured
    - Any free text stating chest pain, ACS, acute coronary syndrome, unstable angina, myocardial infarction, Non-STEMI, or NSTEMI
- Significant ST depression
  - Structured
    - EKG interpretation is present with procedure note documented in the electronic health record.
  - Unstructured
    - EKG is interpreted by the provider in free text or auto-populates in the electronic health record.
- Non-specific repolarization
  - Structured
    - EKG interpretation is present with procedure note documented in the electronic health record.
  - Unstructured
    - EKG is interpreted by the provider in free text or auto-populates in the electronic health record.
- Normal EKG
  - Structured
    - EKG interpretation is present with procedure note documented in the electronic health record.
  - Unstructured
    - EKG is interpreted by the provider in free text or auto-populates in the electronic health record.
- Age
  - Structured
    - Age is always considered structured data.
  - Unstructured
    - Any mention of age in the free text of the electronic health record.
- Risk factors
  - Structured
    - Any past medical history present in the designated section or
    - Any social history present in the designated section of the electronic health record
  - Unstructured
    - Any past medical history or social history typed in the free text sections of the electronic health record.
- Troponin
  - Structured
    - Labs are always considered structured
  - Unstructured
    - Any mention of troponin(s) or cardiac enzyme(s) in the free text of the electronic health record.

**CURB-65**

- Confusion
  - Structured
    - Any documented neurological exam in the designated neurology section or
    - Any documented psychiatric exam in the designated psychiatry portion of the electronic health record or
    - Confusion selected in review of system section of the electronic health record
  - Unstructured
    - Any mention of altered mental status, confusion, or mental status change written in the free text of the electronic health record
- BUN >19mg/dL
  - Structured
    - Labs are always considered structured data
  - Unstructured
    - Any mention of the blood urea nitrogen (BUN) level in the written free text of the electronic health record.
- Respiratory rate > 30
  - Structured
    - Respiratory rate documented in the designated vital signs section of the electronic health records
  - Unstructured
    - Any respiratory rate mentioned or description of breathing rate including tachypnea and bradypnea in the free text sections of the electronic health records.
- SBP<90 or DBP </=60
  - Structured
    - Blood pressure documented in the designated vital signs section of the electronic health records.
  - Unstructured
    - Any blood pressure mentioned or description of blood pressure including hypotension/hypotensive, normotension/normotensive, hypertension/hypertensive in the free text portion of the electronic health record.
- Age
  - Structured
    - Age is always considered structured data.
  - Unstructured
    - Any mention of age in the free text of the electronic health record.
