# Supplementary material for: Assessment of the Feasibility of automated, real-time clinical decision support in the emergency department using electronic health record data
Source: BMC Emerg Med. 2018 Jul 3;18:19. doi: 10.1186/s12873-018-0170-9 (PMC6029277; doi:10.1186/s12873-018-0170-9)
Supplement: Supplementary file 3 — Appendix C. Inclusion and Exclusion of Emergency Medicine Calculators. List of Calculators, Clinical Decision Rules from MDCalc. (DOCX 16 kb) [file 12873_2018_170_MOESM3_ESM.docx]

| **Appendix C: Table C1- Inclusion and Exclusion of Emergency Medicine Calculators** |  |  |  |
| --- | --- | --- | --- |
| **MDCalc Calculator** | **% of total hits** | **Included or Excluded** | **Reason for exclusion** |
| Creatinine clearance cockcroft gault equation | 9.80% | Excluded | Not designed to be CDR |
| Sirs sepsis and septic shock criteria | 7.70% | Excluded | No diagnostic or prognostic significance |
| Wells criteria for pulmonary embolism | 5.99% | Included | Included |
| Thrombolysis in Myocardial Infarction (TIMI) risk score | 4.40% | Included | Included |
| Absolute neutrophil count | 4.31% | Excluded | Not designed to be CDR |
| Corrected QT interval | 3.87% | Excluded | Not designed to be CDR |
| Mean arterial pressure | 3.81% | Excluded | Not designed to be CDR |
| Modified Centor score for Streptococcal A pharyngitis | 3.75% | Included | Included |
| Pulmonary Embolism Rule-out Criteria (PERC) rule | 3.49% | Included | Included |
| Anion gap | 3.37% | Excluded | Not designed to be CDR |
| Sodium correction for hyperglycemia | 2.96% | Excluded | Not designed to be CDR |
| National Institute of Health Stroke Scale (NIHSS) | 2.66% | Excluded | Not designed to be CDR |
| Parkland formula | 2.65% | Excluded | Not designed to be CDR |
| Glasgow Coma Scale score | 2.61% | Excluded | Not designed to be CDR |
| HEART^b^ score for major cardiac events | 2.42% | Included | Included |
| Pediatric Emergency Care Applied Research Network (PECARN) pediatric head injury trauma algorithm | 2.41% | Excluded | <18 yrs old |
| Arterial-alveoli gradient | 2.17% | Excluded | Not designed to be CDR |
| Serum osmolality osmolarity | 2.14% | Excluded | Not designed to be CDR |
| CURB-65^c^ severity score | 2.11% | Included | Included |
| Endotracheal tube size for pediatrics | 1.97% | Excluded | Not designed to be CDR |
| Ransons criteria for pancreatitis mortality | 1.79% | Excluded | Cannot be calculated in enmergency department |
| Wells criteria for DVT | 1.69% | Included | Included |
| ABCD2^d^ score | 1.69% | Included | Included |
| Absolute reticulocyte count index | 1.64% | Excluded | Not designed to be CDR |
| Pneumonia severity index | 1.60% | Included | Included |
| Estimated expected peak expiratory flow | 1.58% | Excluded | Not designed to be CDR |
| Clinical Institute Withdrawal Assessment of Alcohol Scale, Revised | 1.54% | Included | Included |
| ^a^ CDR- clinical decsion rule |  |  |  |
| ^b^History, Electrocardiogram, Age, Risk factors, Troponin |  |  |  |
| ^c^Confusion, Urea, Respiratory rate, Blood pressure, Age ≥ 65 |  |  |  |
| ^d^Age, Blood pressure, Clinical features, Duration of transient ischemic attack |  |  |  |
